# Lessons from a lethal prolongation

**DOI:** 10.1093/ehjcr/ytae086

**Published:** 2024-02-12

**Authors:** Maurits S Buiten, Anastasia D Egorova, Daniela Q C M Barge-Schaapveld, Sebastiaan R D Piers

**Affiliations:** Department of Cardiology, Haaglanden Medical Centre, Lijnbaan 32, 2512 VA, The Hague, The Netherlands; Department of Cardiology, Leiden University Medical Centre, Albinusdreef 2, 2333 ZA, Leiden, The Netherlands; Department of Clinical Genetics, Leiden University Medical Centre, Leiden, The Netherlands; Department of Cardiology, Leiden University Medical Centre, Albinusdreef 2, 2333 ZA, Leiden, The Netherlands

**Keywords:** 5.6 Ventricular arrhythmia, 7.2 Post-cardiac arrest, 5.2 Transient loss of consciousness

## Case description

A 61-year-old male was brought into the emergency department after an out-of-hospital cardiac arrest. The patient’s history consisted of previous heroin abuse, for which he had been taking methadone 120 mg/day for years. Two years prior, the patient had a first syncope, which was adjudicated to be an epileptic seizure. After a second syncope, an implantable loop recorder (ILR) was implanted.

**Figure 1:**
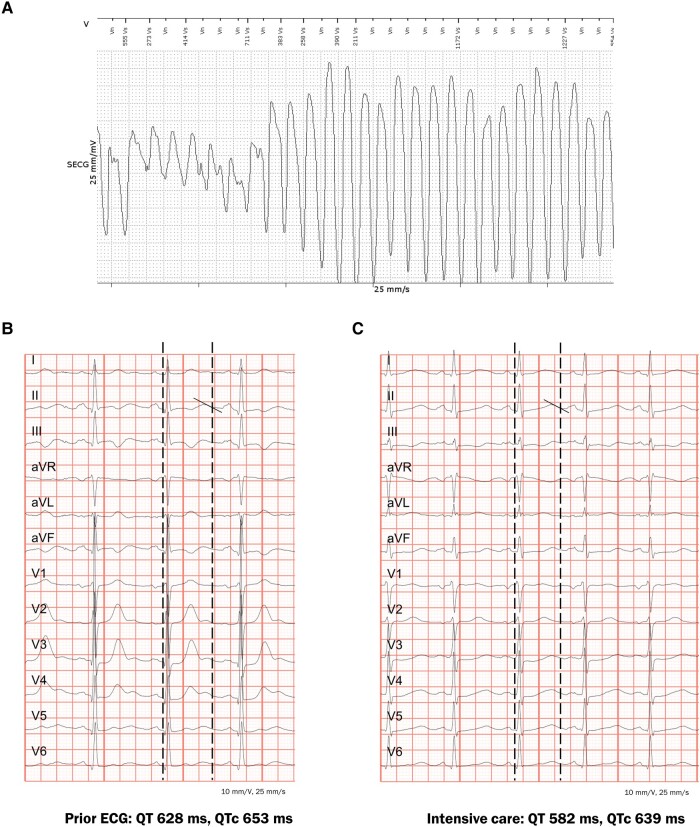
At the time of the cardiac arrest, polymorphic ventricular tachycardia/ventricular fibrillation was registered by the implantable loop recorder (*A*). A prior electrocardiogram (*B*) demonstrated a severely prolonged QTc interval of 653 ms. In leads II and V5, the preferred leads for the measurement of the QT interval, large U-waves can be observed, which were in fact taller than the T-waves and should therefore be included in the QT measurement. The electrocardiogram at the intensive care (*C*) demonstrated a persistent prolonged QTc interval of 639 ms when the potassium was corrected to normal levels.

After waking up, the patient asked for the time and subsequently lost consciousness. The arrest was witnessed by the spouse who alerted emergency services and started basic life support. Ventricular fibrillation was the first rhythm documented, and after a third shock, there was return of spontaneous circulation. The patient was intubated and transported to the emergency department. Blood tests revealed low potassium (2.9 mmol/L, reference 3.5–5.1 mmol/L) and normal magnesium levels. Interrogation of the ILR demonstrated polymorphic ventricular tachycardia (*Figure 1A*). An electrocardiogram (ECG) recorded 2 years previously already showed significant QTc prolongation while patient was on methadone, but this was unfortunately not recognized as such (*Figure 1B*). An ECG pre-dating his methadone use showed no QTc prolongation. The patient was admitted to the intensive care unit, and after correction of the hypokalaemia, additional ECGs confirmed the long QT interval in the absence of any QT-prolonging drugs other than methadone (*Figure 1C*). Unfortunately, electroencephalography demonstrated absent electrical brain activity, and the patient died after termination of supportive treatment. At autopsy, no structural heart disease was found, and genetic testing did not reveal any long QT syndrome (LQTS)–related mutation.

Most likely, the patient had acquired LQTS due to the combination of methadone use and low potassium levels. This case emphasizes the importance of correct measurement of the QT interval as well as the need to be aware of QT prolongation in methadone use. Methadone is a synthetic opioid that blocks IKr and may thereby result in QT prolongation and a significant risk of torsade des pointes.^1^ In collaboration with the Heart Rhythm Society, the American Pain Society recommends follow-up ECGs for QTc monitoring at methadone doses ≥ 30 mg/day.^2^

**Consent:** The patient’s relatives provided written consent for publication of this case.

**Funding:** None declared.

## Data Availability

The data underlying this article are available within the article.
